# NOBEL-BOX: Development of a Low-Cost Ship-Based Instrument for Ocean Monitoring

**DOI:** 10.3390/s23249654

**Published:** 2023-12-06

**Authors:** Noir P. Purba, Ibnu Faizal, Marine K. Martasuganda, Ajeng Wulandari, Rd. Salsa D. Kusuma, Muhammad H. Ilmi, Choerunnissa Febriani, Raffy R. Alfarez, Fickry Argeta, Jati S. Wicaksana

**Affiliations:** 1 Department of Marine, Faculty of Fishery and Marine Science, Universitas Padjadjaran, Jl. Raya Bandung-Sumedang Km. 21, Jatinangor 45363, Indonesia; ibnu.faizal@unpad.ac.id; 2 Jack Dn’t Swim, Bandung 40266, Indonesia; ajengwulandari74@gmail.com (A.W.); salsadwksm@gmail.com (R.S.D.K.); 3 Movement for the Ocean (MOCEAN) Foundation, Jl. Batununggal Indah Raya no. 199 Batununggal, Bandung 40267, Indonesia; marine.kenzi@gmail.com; 4 KOMITMEN Research Group, Universitas Padjadjaran, Jl. Raya Bandung-Sumedang Km. 21, Jatinangor 45363, Indonesia; muhammad19067@mail.unpad.ac.id (M.H.I.); choerunnisa19001@mail.unpad.ac.id (C.F.); raffy20001@mail.unpad.ac.id (R.R.A.); fickry.argeta18@gmail.com (F.A.); 5 Basic Inter Teknologi Intersolusi, Bandung 41563, Indonesia; jsatriaw@gmail.com

**Keywords:** ocean observation, low-cost instrument, water quality, Indonesia seas, ocean health

## Abstract

This research aims to develop an inexpensive ocean observation instrument with the project name NOBEL (Nusantara Oceanography Backdoor Experiment Laboratory)-BOX. The device can be installed on all types of vessels for mapping the water conditions, providing accurate data for managing a marine area, particularly regarding water quality. The principle of NOBEL-BOX is to attach six sensors in a container connected to a microcontroller and then measure specific data directly and automatically. The methodology employed included experimental design, laboratory and field tests, and data evaluation to develop the necessary system and instruments. The design process encompassed the construction of the instrument and the fabrication, involving the creation of three-dimensional drawings and the design of microcontrollers and data transmission systems and power capacity. This instrument is box-shaped with a microcontroller, sensors, a battery, and cables located inside. The testing phase included data validation, testing of the device in the laboratory, and field testing showed that the device worked. The data provided from this instrument could meet the specific criteria for seawater analysis.

## 1. Introduction

As an archipelagic country, the Indonesian seas located along the equator have complex and unique characteristics. Several factors that lead to its dynamics include monsoon patterns, bathymetry, and the ocean circulation. These dynamics result from the interaction between two oceans, the Indian and the Pacific Ocean, and two continents, the Asian and Australian continents [1,2,3]. Consequently, these circulations transport various materials, such as nutrients that support marine biota and coastal ecosystems [4,5]. However, harmful materials such as heavy metals and marine debris, which pose a serious threat in Indonesian waters, are also transported from rivers, including those originating from other surrounding countries [6,7]. Due to multiple challenges in Indonesian seas, the Ocean Health Index (OHI) score for Indonesia is 63 out of 100, which is below the global average of 69 [8].

In many countries, the need for monitoring instruments, data, and systems is a key strategy for the sustainable management of these regions, including prediction and mitigation. However, the lack of infrastructure, human resources, data coordination, and sharing and data gaps are still issues [9,10]. Recently, monitoring the condition of the ocean has become a pressing concern in the global scientific community [11]. Various efforts and methods have been attempted to develop innovative technologies for monitoring marine dynamics and its contents [12]. The availability of data collected and inventoried from high-precision technologies will be the basis for understanding physical and biological marine processes, especially in the climate change era [13,14,15]. However, conventional methods of ocean monitoring mainly still being carried out through direct methods come with challenges, including high costs, limited agencies focusing on marine observation, and the inherent complexity of natural ocean characteristics [16]. 

To address these challenges, there has been a recent revolution in ocean-observing technology, enabling new and effective ways to observe and monitor the ocean [17]. Recently, several low-cost marine instruments have been developed, aiming to provide extensive data with cheaper cost of production [18]. For instance, a drifter has been successfully designed and tested for developing countries [16], while [12] developed portable water-quality sensors and successfully tested them in open seas. Another development was achieved using wireless sensors network technology to study water quality [19]. However, its function, reliability, and accuracy still need to be enhanced and many of the low-cost instrument developments have encountered difficulties compared with the reference sensors and their accuracy [18].

In this context, a novel ocean monitoring instrument, NOBEL-BOX, was designed by integrating IoT (Internet of Things) with the low-cost probes. This instrument was designed to be a cost-effective solution and offers a budget-friendly option for accurate measurements and a small size that can be installed on various type of ships, reducing operational costs and the need for extensive human resources. This approach has already been implemented in European countries, especially for installation on ferries, with a cost of around EUR 50,000 [20,21]. Furthermore, this instrument has been able to produce data that can map several phenomena, such as upwelling [22,23], water-quality monitoring [19,24], and carbon sink analysis [25]. Previous findings, for instance, in Indonesia, with the concept of low-cost ocean monitoring instruments have been successfully applied with the RHEA and ARHEA instruments [26,27]. With their proven track record in delivering accurate technical data, these new instruments represent a significant advancement in covering Indonesian seas and can be applied in other seas within the ocean monitoring system. As a result, these instruments offer a valuable tool for decision-makers and governments in archipelagic countries, specifically for Indonesian seas management [28]. 

NOBEL-BOX is designed to measure multiple parameters in near real time, including sea and atmosphere temperature, salinity, dissolved oxygen (DO), total dissolved solid (TDS), and pH, using the modified Arduino UNO microcontroller system. These parameters collectively offer valuable insights into the state of marine environments, enabling research on climate change, ocean circulation, water quality, ecosystem health, and the impact of human activities on the world’s oceans. This novel instrument is intended to be cost-effective and suitable for various types of ships, such as fishing boats, cargo ships, and ferries, facilitating measurements without requiring specific research vessels. Indonesia, with approximately 572,000 fishing vessels and 63,000 commercial ships navigating its waters, has a substantial maritime presence. Given the vast amount of sea transportation in Indonesia, it is expected that this instrument will be embedded and operational under all water conditions, providing accurate technical data to determine fishing zones, assess water pollution levels, support parameters for marine spatial zoning, and monitor water quality throughout the country. The data generated by NOBEL-BOX can contribute significantly to defense aspects, fisheries, and marine mitigation and government regulation, aligning with Sustainable Development Goals target 13 (climate change) and target 14 (life below water). 

## 2. Materials and Methods

Our concepts is to build NOBEL-BOX as an instrument that is compact, easily transportable, and capable of providing high-quality data in marine operations. Considering these requirements, the following design needs were obtained: the size of the vessel was made as small as possible to allow mobilization and deployment by one or two personnel; low-cost, good charge/discharge capability and battery capacity, and good mission duration, where the ability to carry out a mission for five years, were also determined as important factors. 

The development of the low-cost NOBEL-BOX consisted of three processes. It encompassed various stages, including experimental design, laboratory and field tests, and data assessment. The design phase involved intricate steps such as constructing the instrument and fabricating the device. This process entailed the generation of detailed three-dimensional sketches and drawings, the development of microcontrollers, and the design of data transmission systems, all while carefully determining power capacity to ensure optimal functionality.

The parameters were chosen through a literature review, which is essential for ocean observation [18,27,29,30]. The sensors in the literature include those for surface water temperature, air temperature, salinity, DO, pH, and TDS. In the subsequent testing phase, rigorous measures are undertaken to ensure the device’s accuracy and reliability. This phase comprises data validation, meticulous testing within controlled laboratory environments, and comprehensive field testing under real-world conditions. The outcomes of both laboratory and field tests affirm the device’s effectiveness, as it consistently delivers high-quality data suitable for conducting in-depth spatial and temporal analyses of seawater conditions.

### 2.1. System Description

**Figure 1:**
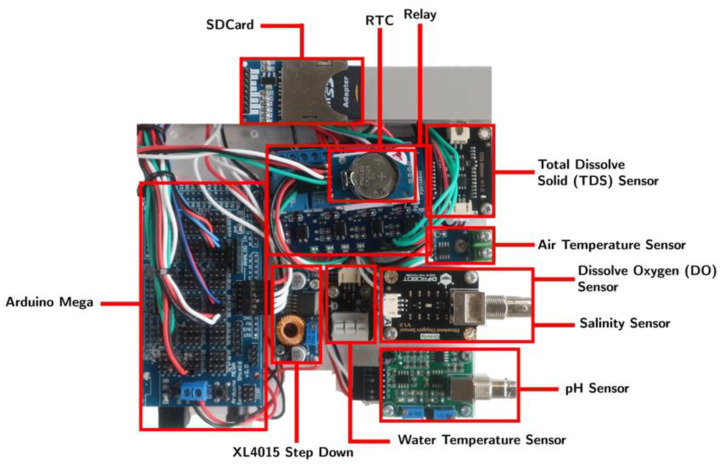
Main components, connection, and probes connector.

The principle of NOBEL-BOX is to combine several probes, including ones to determine seawater temperature, air temperature, pH, salinity, TDS, and DO. These probes are connected to a microcontroller. A pump system was added to facilitate the supply of seawater samples measured by this instrument. In general, this instrument is shaped like a box with the dimensions of 30 × 40 × 20 cm with a total weight, including when all components are assembled within the housing, of around 15 kg. With this weight and dimension, it is hoped that this instrument can be placed on various ships, including small wooden ships. This instrument can automate self-cleaning by spraying pure water onto the probes to minimize data bias from previous measurements. Furthermore, the measurement data are stored on a micro-SD card that can be transferred to a desired server using a mobile communications (GSM) system. The instrument system operation device uses Arduino Uno R3 and Arduino Mega 2560 microcontrollers manufactured by Arduino, Turin-Italy (Figure 1). 

**Figure 2:**
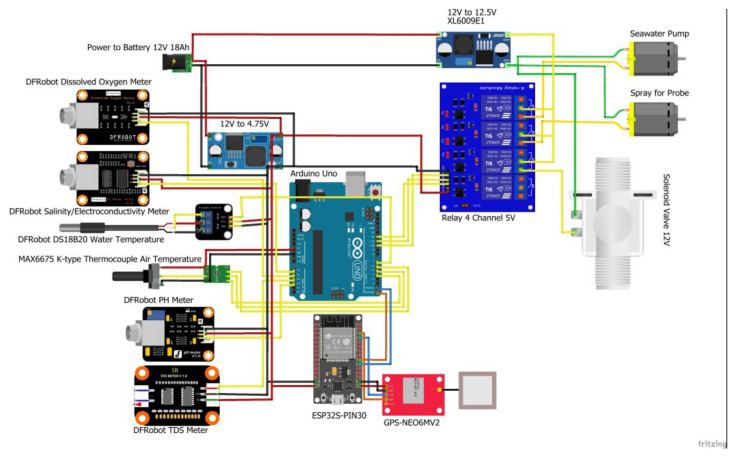
Electronic schematics including sensors, Arduino Uno, bateray and seawater sampling. Red cables represent positive wires, and black cables represent negative wires. Other colours represent the line connections (Please refer to Table 1 for information about manufacturers).

The microcontroller is assembled through the Arduino Mega 2560 CPU Board manufactured by Arduino, Turin-Italy [31] as the central system, equipped with other components such as the XL4015 Step Down manufactured by XLSEMI, Shanghai-China [32] to reduce the voltage; a solid-state relay [33] to automate the water pump, sample measurement, and solenoid automation regularly; DS3231 RTC [34] to display the real-time clock; a MicroSD Module [35]; and an SD Card to store data. It also has modules measuring seawater, such as TDS, pH, water temperature, atmospheric temperature, salinity, and DO. It has an LCD [36] to display real-time measurement data and clocks (Figure 2).

An ACCU/battery is used to power the instrument. Other components, including a seawater pump and a freshwater pump for sensors cleaning after sampling, are attached within the housing. The NOBEL-BOX is equipped with a small shelf for easy replacement of the ACCU when it is no longer functional, making it easier to remove the microcontroller in case of a short circuit. The seawater tank with a built-in sensor is not permanently attached and designed to be easily replaceable. It features a rubber-equipped hole to prevent water leakage and facilitate sensor maintenance. The clean water tank is designed to be easily refilling, with a an upward-extending pipe that enables filling without opening the box. Additionally, to maximize usage, a non-conductive separator must be placed between the sensors or limit the sensor’s range. This separation is necessary, as the sensor emits electrical energy, and isolating it ensures accurate sensor readings without interference from other sensors.

### 2.2. Sensors and Calibrations

All the sensors should meet the specific criteria for ocean measurements. To ensure this instrument can effectively operate over long periods, the sensors were chosen based on the previous developments by [28,37]. Most recent publications on the data collected from these sensors show that they meet the necessary quality and reliability standards for the intended purpose [18]. These sensors also offer the best quality in the online market. The characteristics of the sensors can be found in Table 1.

**Table 1:** The sensor components used in NOBEL-BOX.

Component	Accuracy/Range	Working Range	Time to Recalibrate	Average Life of Probe	Manufacturer
Gravity: DS18B20 Temperature Sensor (DS18B20)	±0.5 °C	−10 °C to +85 °C	N/A	N/A	DFRobot, Shanghai-China
Thermocouple Temperature Sensor K-Type (MAX6675)	±0.25 °C	−20°to +80°	N/A	N/A	Maxim Integrated Product, CA, USA
Gravity: Analog Dissolved Oxygen Sensor (SEN0237)	±0.05 mg/L	0 to 20 mg/L	~1 year	>0.5 year	DFRobot, Shanghai-China
Sensor TDS Meter V1.0 for Arduino Analog TDS (XH2.54-3P)	±10% F.S. (25 °C)	0 to 1000 ppm	N/A	>0.5 year	DFRobot, Shanghai-China
Module Analog P.H. Sensor Kit PH-4502C Electrode Probe (PH-4502C)	±0.1 P.H.	0 to 14	~1 year	>0.5 year	DFRobot, Shanghai-China
Gravity: Analog Electrical Conductivity Sensor Meter v2 K = 10 (EP000533)	±5% F.S.	0 to 20 ms/cm	N/A	>0.5 year	DFRobot, Shanghai-China

During the trial, both in freshwater tanks and in the ocean, the sensors installed in the NOBEL-BOX were compared with data from other portable sensors. For salinity, an Atago hand refractometer; for DO, a YSI Pro20 Dissolved Oxygen Meter Pro 20; and for temperature and pH, a HOBO Bluetooth Low Energy and Temperature Logger was used. These kinds of instruments have been widely used for measurement ocean conditions and studying complex ecosystems [38,39,40,41,42,43]. The testing process involved data validation and testing of the instrument in both laboratory and field environments.

The sensors were calibrated with a buffers pack following the manufacturer’s guidance. DO was measured by obtaining voltage values and corresponding DO levels. Next, a linear graph was created, and error, accuracy, and standard error were assessed. Additionally, consideration was given to compensating for DO values by incorporating temperature data. Next, we calibrated the TDS meter using the same method as for calibrating the DO sensor. For the pH sensor, two buffers were provided at pH 4 and 7, facilitating calibration by aligning voltage references with their respective pH values. This process yielded the actual pH values for buffers at pH 4 and 7. The calibration of the electrical conductivity sensor followed a similar procedure, using a standard buffer solution of 12.88 ms/cm to match voltage references with EC values. Subsequently, EC values were converted into salinity. As for the temperature sensors, they did not require calibration since they are digital sensors, eliminating the need to convert voltage into values. According to the manufacturer’s instructions, recalibration is recommended at intervals exceeding 0.5 years. The manufacturers recommend routine sensor maintenance, including maintaining sensors free from dirt deposits and leakage to ensure optimal functionality. Furthermore, based on previous findings, the sensors will need to be recalibrated again around one year to maintain the accuracy, except for temperature sensors, both air and water, which do not need recalibration [18].

### 2.3. Operations Instructions

**Figure 3:**
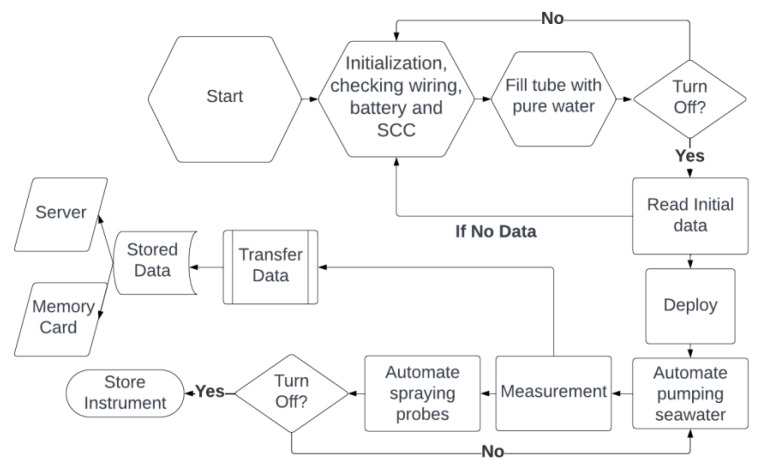
Step-by-step operation NOBEL-BOX.

Before installation on the vessel, inspections of data storage, battery capacity, and LED functionality must be conducted. During field testing, this instrument stores data on a memory card and can transmit data via a GSM signal, adapting to varying data conditions and requirements. The stored data are in CSV format and can be opened with various simple applications for visualization. In essence, NOBEL-BOX is designed to be adaptable for use on all types of vessels and in various water conditions, ensuring the generation of accurate data even in complex water environments. The step-by-step guide can be seen in Figure 3.

In the field trial, the NOBEL-BOX can be configured to measure based on the monitoring objectives. In this project, once the system is activated, it operates in a cycle to measure water quality every ten minutes. The seawater sample measurement is taken after the measurement tube is filled with the sample. The system will turn on and prepare for three minutes, followed by a 3.5 s seawater-pumping phase. Subsequently, the sensor will measure the collected seawater in the tank for two minutes. The solenoid then opens to dispose of the measured seawater sample. Afterward, the clean water pump runs for 3.5 s to cleanse any remaining seawater clinging to the tube and sensors. The clean water is retained for one minute before the solenoid opens to discharge the water, and the cycle starts again. It is crucial to note that if water leakage occurs and causes the pump or microcontroller to short circuit, attention should be paid to the wave height and thus the water that can enter the box. 

## 3. Results

### 3.1. Final Design

**Figure 4:**
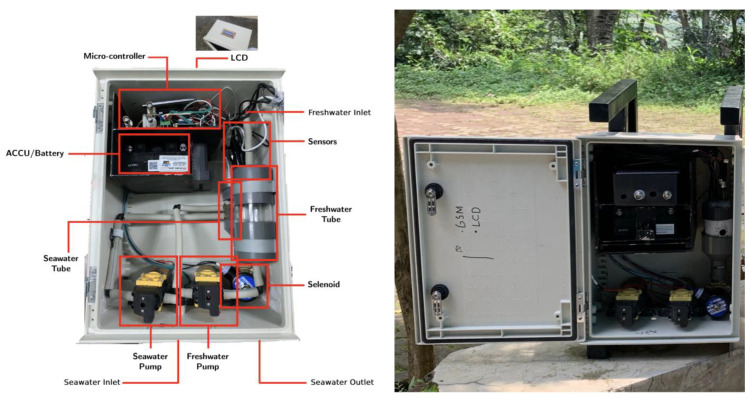
Inside NOBEL-BOX with housing (**left**) and supporting black frame on the back (**right**). Please refer to Table 1 for information about manufacturers.

The final design of the NOBEL-BOX takes the form of a compact and practical box shape, which was sourced from online markets. This box-like structure is complemented by a support frame at the back, enhancing durability and stability during installation and operation for sea conditions (Figure 4). The integrated frame not only adds robustness to the design but also contributes to its adaptability for placement on various vessels, including smaller ships.

This box is made of fiber material, making it resistant to water splashes. On the inside, there are rubber partitions to prevent water from entering the housing. Subsequently, the placement of the solenoid, freshwater tube, and seawater sensors tube are positioned on the right side, while the microcontroller and battery are positioned on the upper left side. Below, there are pumps for seawater and freshwater. The freshwater pump serves the purpose of cleaning the sensors after measurements are taken. In other sections, cables and pipes are present. Freshwater is introduced from the top side, while a pipe is situated on the lower right side. At the back, there is a supporting frame designed to adjust the positioning of the NOBEL-BOX according to the ship’s conditions, enabling easy installation. Furthermore, during maintenance, the supporting frame can be detached from the main housing, simplifying transportation. This supporting frame can be replaced or adjusted in shape to accommodate the characteristics of the ships.

### 3.2. Testing

**Figure 5:**
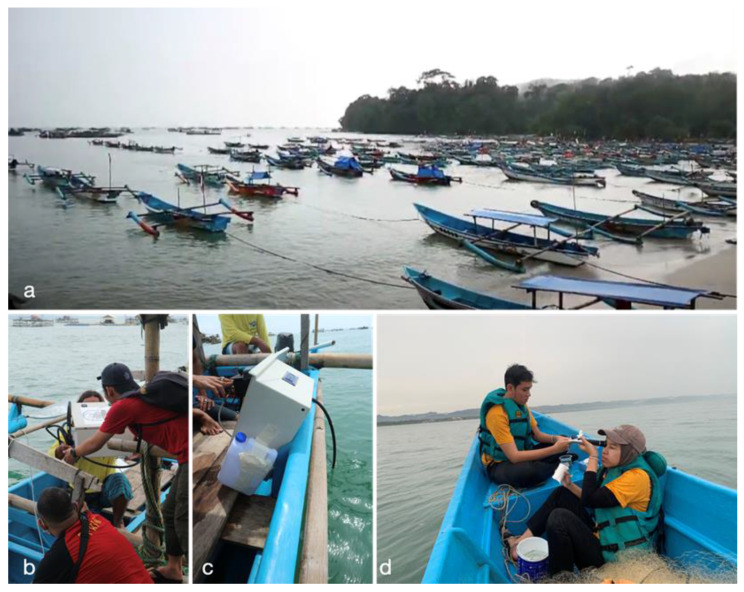
Sea trial in Pangandaraan beach, (**a**) sea condition during the test and type of wooden boat used, (**b**) team and NOBEL-BOX mobilization, (**c**) seawater pumped into the instrument (black water hose), and (**d**) measurements using other portable instruments at the ship’s bow.

NOBEL-BOX has been tested in the freshwater ponds, laboratory, and the east coast of Pananjung Village, Pangandaraan District, West Java. In freshwater ponds, tests were conducted to assess the instrument’s capabilities in performing its functions. Subsequently, in the laboratory, tests were carried out by examining the data generated through sensor responses to changes in water conditions. This was achieved by mixing seawater with freshwater at various volume differences. The final test (sea trial) was conducted in Pangandaraan Bay, located in southwestern Java. This area is characterized by a marine protected area (MPA), tourism, and fisheries activities around the bay. The waters directly face the Indian Ocean, which has strong ocean currents and high waves [42] (Figure 5). 

The NOBEL-BOX instrument was tested for two days (6 to 7 December 2022) under cloudy and lightly rainy weather conditions. The instrument was mounted on a 5 GT wooden fishing boat, and measurements were taken along the west coast of Pangandaraan at pre-determined stations. The primary objective of the test was to thoroughly evaluate the performance and functionality of the sensors integrated into the device. Throughout the course of this assessment, a distinct configuration was adopted: the length of the seawater pump-pipe was precisely set at two meters, while the device’s placement relative to the surface of the water was consistently maintained at a depth of 0.5 m.

### 3.3. Data Analysis

**Figure 6:**
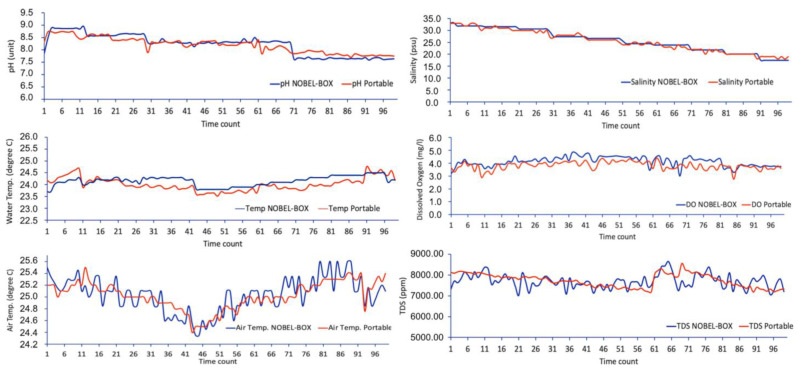
NOBEL-BOX data results compared to other portable devices during laboratory and field tests.

The data results obtained from NOBEL-BOX compared to those of other portable devices, both in laboratory and field test settings, provided time-series data (Figure 6). The data obtained can be processed using M.S. Excel or Ocean Data View (ODV) software to be presented as graphs to observe temporal or spatial data [43]. 

Based on the TDS sensor datasheet, the device has an accuracy of approximately 90% at a temperature compensation of 25 °C. However, this can be minimized by reducing data drift in the Arduino IDE, which can improve the accuracy up to 95%. As for the pH sensor, it has an accuracy of 98% based on a temperature compensation of 25 °C, as indicated in the datasheet. Attempts were made to reduce data drift for pH, but there was no significant improvement, so the accuracy remained at 98%. The water temperature data resolution of up to 0.5 °C due to temperature measurement was not calculated, making it difficult to reduce data drift for better resolution. Regarding the DO sensor, it showed an error range of 1–1.5%, with the note that data drift reduction was applied through the Arduino IDE, resulting in data accuracy of up to 99%. The salinity sensor also showed an error range of up to 5% with temperature compensation at 25 °C, which was reduced to 2% by minimizing data drift through calculations in the Arduino IDE. The air temperature sensor showed a resolution of 0.25 °C based on the datasheet. The reduction of data drift was achieved by taking 10,000 data points from each sensor, which were then be used to calculate the average or median (for the TDS sensor), resulting in more stable and less significant data drift. 

## 4. Discussion

The electronic system that was built was shown to function properly. In the experiments conducted both in controlled pool conditions and out in the sea, all components functioned without any issues, and the installed sensors successfully generated data (Figure 1, Figure 2 and Figure 3). Previous findings have stated that numerous low-cost marine instruments utilizing Arduino Uno have consistently demonstrated the effectiveness of this system [29,44,45]. 

The housing of the NOBEL-BOX instrument is exceptionally versatile, securely housing all the sensors within the enclosure (Figure 4). This design not only ensures the safe transport of the equipment but also facilitates its seamless integration onto various platforms, including wooden ships. Many previous findings have utilized a similar box design, even though the instruments come in different sizes [46]. In general, these low-cost water-quality instruments share a common feature of being housed within a box. This box is designed to be waterproof and withstand a certain level of pressure, making it user-friendly. The box-shaped design allows it to adapt to the shape of the vessel with the addition of a frame at the rear. During the sea trial, despite the presence of waves that caused the ship to sway, the instrument remained undamaged and fully operational. Furthermore, based on the design concept acquired directly from online markets, it appears that all the components can fit neatly into the housing, including the cables and other things. However, during the evaluation process, attention was drawn to the arrangement of the cables and pipes, which need to be more organized at the rear. Furthermore, the weight of the NOBEL-BOX instrument is suitable to be lifted by a single person. This is important due to it is alignment with the concept of man-portable instrument (Figure 5b). 

Selecting the optimal location for the instrument placement is vital to ensure the integrity of the data it collects. The optimal positioning of this instrument is alongside the ship (Figure 5c). Placing the instrument at the rear of the ship could result in the seawater being stirred up by the ship’s propellers, leading to compromised data accuracy due to turbulence. If the instrument were to be placed directly behind the ship, the measurements it takes could be influenced by these disturbances, leading to inaccuracies in the collected data. By positioning the instrument alongside the ship, it remains in a relatively undisturbed flow of water. This minimizes the potential for mixing and turbulence, allowing the sensors to capture a more representative and precise snapshot of the seawater conditions. Consequently, the data collected would be more reliable and reflective of the actual environmental parameters.

The battery installed in this instrument has the ability to charge or discharge with sufficient battery capacity, allowing it to carry out its mission for up to five years with calibration every year. The battery embedded in the NOBEL-BOX has the ability to monitor battery voltage. It can be directly observed with a battery indicator scale ranging from 0 (low battery) to 4 (full battery) bars. The battery consumption depends on the data collection time and length of the seawater pump. In the sea trial, the length of the pipe measured approximately two meters (Figure 5c). It is important to note that as the length of the pipe increases, a corresponding effect emerges: The duration for which the battery remains operational also increases. This correlation between pipe length and battery lifespan is attributed to the intricacies of the system’s energy consumption. As a result, the battery is able to power the instrument for an extended period before necessitating recharging or replacement. This further underscores the importance of tailoring the instrument’s configuration to meet the specific demands of its application environment.

Comparing the data results obtained from NOBEL-BOX to those of other portable devices, both in laboratory and field test settings, provides valuable data to assess its performance and capabilities (Figure 6). This assessment allows us to gauge the effectiveness and reliability of NOBEL-BOX as a data collection tool in diverse environments. To this point, the sensors have shown promising results in capturing the measured oceanographic parameters, such as temperature, salinity, and pH (Figure 6 and Table 2). In the specific case, there was a spike in DO data due to slower response time than the other sensors. The sensors have complex formulas and have a sampling rate to obtain good accuracy and minimize errors. Previous finding stated that the mismatch between the sampling frequencies of sensors is a common issue in machine monitoring applications [47]. The sensors have more complex formulas in the Arduino IDE, affecting the sensitivity of the data. Sometimes, measurements are conducted more rapidly to observe the instrument’s response, indicating that DO sensors may require additional time to complete accurate measurements.

## 5. Conclusions

It can be concluded that NOBEL-BOX is a necessary marine instrument due to its efficiency and economic benefits. Therefore, the development of the prototype was designed to be compact in size, man-portable, and act as a survey platform capable of providing high-quality data in marine operations. While this initial design instrument does not possess waterproof and buoyancy features, several assessments will be a top priority in the subsequent development stages. The incorporation of water resistance and buoyancy capabilities will be addressed through rigorous testing and adherence to international standards to ensure the device’s suitability for marine applications. This instrument integrated software including built-in knowledge of hardware integration, including creating user friendly systems, tests, measurements, and data. Although NOBEL-BOX provides the tools required for most ocean measurements, it is an open development environment. In the future, standardization of housing and safety, measurement and control hardware, and open standards, which define interoperability between multiple areas of expertise, can be improved.

The data generated by this instrument allow for monitoring from semi-enclosed waters to open ocean. Considering the Indonesian area as an archipelagic country with vast seas, this instrument provides the benefit of observing external factors that may disturb the ecosystem and biota. The NOBEL-BOX instrument is a cost-effective solution for mapping marine conditions in a specific area with high data precision. It provides accurate and real-time data for effective marine management. The design process involved the creation of a compact and portable device that can be easily installed and used on all types of ships. Overall, this research presents a valuable contribution to oceanography, providing a cost-effective solution for real-time monitoring of marine conditions.

## 6. Patents

This instrument has been submitted for patent application under the reference number DID2023030194, bearing the name “NOABOX” and accompanied by its corresponding logo, on the date of 11 April 2023 (https://pdki-indonesia.dgip.go.id/detail/5317488cad5b0c89f985829decf2dcbef8c05347d8db02c91a7c5e8873c0ae33%3Fnomor=IPT2023061466?type=trademark&keyword=noabox) (accessed on 17 August 2023).

## Figures and Tables

**Table 2:** Data statistics from each parameter.

	pH (Unit)		Water Temp. (°C)		Air Temp. (°C)		Salinity (psu)		TDS (ppm)		DO (mg/L)	
	N.B.	PO	NB	PO	NB	PO	NB	PO	NB	PO	NB	PO
Average	8.219	8.197	24.013	24.156	25.036	25.028	25.698	25.495	7700.255	7739.192	4.126	3.805
Min.	7.590	7.700	23.510	23.700	24.350	24.400	17.500	18.000	7027.400	7187.000	3.000	2.750
Max.	8.950	8.750	24.760	24.500	25.600	25.500	33.240	33.000	8658.760	8565.000	4.830	4.380

## Data Availability

The data that support the findings of this study are available on request from the corresponding author.

## References

[1] Guo Y., Wang L.F. (2023). Destinations and pathways of the Indonesian Throughflow water in the Indian Ocean. J. Clim..

[2] Katavouta A., Polton J.A., Harle J.D., Holt J.T. (2022). Effect of Tides on the Indonesian Seas Circulation and Their Role on the Volume, Heat and Salt Transports of the Indonesian Throughflow. J. Geophys. Res. Ocean..

[3] Scroxton N., Gagan M.K., Ayliffe L.K., Hantoro W.S., Hellstrom J.C., Cheng H., Edwards R.L., Zhao J.-X., Suwargadi B.W., Rifai H. (2022). Antiphase response of the Indonesian–Australian monsoon to millennial-scale events of the last glacial period. Sci. Rep..

[4] Xie T., Newton R., Schlosser P., Du C., Dai M. (2019). Long-Term Mean Mass, Heat and Nutrient Flux through the Indonesian Seas, Based on the Tritium Inventory in the Pacific and Indian Oceans. J. Geophys. Res. Oceans.

[5] Taufiqurrahman E., A’an J.W., Masumoto Y. (2020). The Indonesian throughflow and its impact on biogeochemistry in the Indonesian Seas. ASEAN J. Sci. Technol. Dev..

[6] Adyasari D., Pratama M.A., Teguh N.A., Sabdaningsih A., Kusumaningtyas M.A., Dimova N. (2021). Anthropogenic impact on Indonesian coastal water and ecosystems: Current status and future opportunities. Mar. Pollut. Bull..

[7] Purba N.P., Faizal I., Cordova M.R., Abimanyu A., Afandi N.K., Indriawan D., Khan A.M. (2021). Marine Debris Pathway Across Indonesian Boundary Seas. J. Ecol. Eng..

[8] Halpern B.S., Frazier M., Afflerbach J., O’hara C., Katona S., Lowndes J.S.S., Jiang N., Pacheco E., Scarborough C., Polsenberg J. (2017). Drivers and implications of change in global ocean health over the past five years. PLoS ONE.

[9] Révelard A., Tintoré J., Verron J., Bahurel P., Barth J.A., Belbéoch M., Benveniste J., Bonnefond P., Chassignet E.P., Cravatte S. (2022). Ocean Integration: The Needs and Challenges of Effective Coordination within the Ocean Observing System. Front. Mar. Sci..

[10] Xavier F.N.d.C., Martins L.D., Oyamada M.S., Spanhol F.A., Coutinho F.R., Pfrimer F.W.D., de Camargo E.T. (2022). Evaluation of low-cost sensors for real-time water quality monitoring. Anais Estendidos do XII Simpósio Brasileiro de Engenharia de Sistemas Computacionais.

[11] Butler J., Pagniello C., Florida International University (2021). Emerging, Low-Cost Ocean Observing Technologies to Democratize Access to the Ocean. Oceanography.

[12] Marcelli M., Piermattei V., Madonia A., Mainardi U. (2014). Design and Application of New Low-Cost Instruments for Marine Environmental Research. Sensors.

[13] Roemmich D., Johnson G., Riser S., Davis R., Gilson J., Owens W.B., Garzoli S., Schmid C., Ignaszewski M. (2009). The Argo Program: Observing the Global Oceans with Profiling Floats. Oceanography.

[14] Liu Y., Qiu M., Liu C., Guo Z. (2016). Big data challenges in ocean observation: A survey. Pers. Ubiquitous Comput..

[15] Levin L.A., Bett B.J., Gates A.R., Heimbach P., Howe B.M., Janssen F., McCurdy A., Ruhl H.A., Snelgrove P., Stocks K.I. (2019). Global Observing Needs in the Deep Ocean. Front. Mar. Sci..

[16] Gerin R., Zennaro M., Rainone M., Pietrosemoli E., Poulain P.M., Crise A. (2018). On the design of a sustainable ocean drifter for developing countries. EAI Endorsed Trans. Internet Things.

[17] Wilson T., Barth J., Pierce S., Kosro P., Waldorf B. A Lagrangian drifter with inexpensive wide area differential GPS positioning. Proceedings of the OCEANS 96 MTS/IEEE Conference Proceedings, The Coastal Ocean—Prospects for the 21st Century.

[18] de Camargo E.T., Spanhol F.A., Slongo J.S., da Silva M.V.R., Pazinato J., Lobo A.V.d.L., Coutinho F.R., Pfrimer F.W.D., Lindino C.A., Oyamada M.S. (2023). Low-Cost Water Quality Sensors for IoT: A Systematic Review. Sensors.

[19] Demetillo A.T., Japitana M.V., Taboada E.B. (2019). A system for monitoring water quality in a large aquatic area using wireless sensor network technology. Sustain. Environ. Res..

[20] Kuznetsov A.S., Shapovalov Y.I., Shapovalov R.O. (2019). Results of Monitoring the Surface Fields Dynamics in the Black Sea Waters Using a Ferry Box System. Phys. Oceanogr..

[21] Petersen W. (2019). Innovative Sensor Carriers for Cost-Effective Global Ocean Samplings-Platforms of opportunity in action: The FerryBox system. Challenges and Innovations in Ocean In Situ Sensors-Measuring Inner Ocean Processes and Health in the Digital Age.

[22] Irion R. (1998). Ocean Scientists Find Life, Warmth in the Seas. Science.

[23] Langis D.P. (2015). Arduino Based Oceanographic Instruments: An Implementation Strategy for Low-Cost Sensors.

[24] Gunia M., Laine M., Malve O., Kallio K., Kervinen M., Anttila S., Kotamäki N., Siivola E., Kettunen J., Kauranne T. (2022). Data fusion system for monitoring water quality: Application to chlorophyll-a in Baltic sea coast. Environ. Model. Softw..

[25] Macovei V.A., Petersen W., Brix H., Voynova Y.G. (2021). Reduced Ocean Carbon Sink in the South and Central North Sea (2014–2018) Revealed from FerryBox Observations. Geophys. Res. Lett..

[26] Zenyda K.S., Subiyanto, Faizal I., Prayogo N., Purba N.P. (2021). Evaluation of a New Integrated Marine Instruments: RHEA (Drifter GPS Oceanography Coverage Area). IOP Conf. Ser. Earth Environ. Sci..

[27] Purba N.P., Faizal I., Mulyani P.G., Prayogo N., Prasetyo T., Khan A.M. (2019). Performance of lagriangan drifter oceanography coverage area (RHEA): Second phase. Int. J. Ocean. Oceanogr..

[28] Purba N.P., Faizal I., Valino D.A., Kang H.S., Sugianto E., Martasuganda M.K., Abimanyu A., Bratasena T., Zenyda K.S., Prayogo N. (2023). Development of autonomous multi-sensor ocean monitoring instrument designed for complex archipelagic waters. Int. J. Environ. Sci. Technol..

[29] Hakimi I.M., Jamil Z. (2021). Development of Water Quality Monitoring Device Using Arduino UNO. IOP Conf. Ser. Mater. Sci. Eng..

[30] Rao A.S., Marshall S., Gubbi J., Palaniswami M., Sinnott R., Pettigrovet V. Design of low-cost autonomous water quality monitoring system. Proceedings of the 2013 International Conference on Advances in Computing, Communications and Informatics (ICACCI).

[31] Appiani A. (2022). Arduino^®^ MEGA 2560 Rev3 Features. Arduino^®^ MEGA 2560. Lex Russ..

[32] XLSEMI *Datasheet Buck DC to DC XL4013*; 2015, pp. 1–10. www.xlsmi.com.

[33] Technology H. (2015). User Guide—4 Channel 5V Optical Isolated Relay Module. Occup. Health Saf..

[34] Maxim Integrated *DS3231 RTC General Description*; 2015; p. 20. www.maximintegrated.com.

[35] EBay *Micro SD Card Card Adapter Reader Module for Arduino*; 2013; pp. 1–2. https://www.ebay.com/itm/225559844980.

[36] Hitachi *HD44780U (LCD-II)*; 2015; Volume 3304, pp. 1–19. https://html.alldatasheet.com/html-pdf/63663/HITACHI/HD44780U/247/1/HD44780U.html.

[37] Adriman R., Fitria M., Afdhal A., Fernanda A.Y. An IoT-Based System for Water Quality Monitoring and Notification System of Aquaculture Prawn Pond. Proceedings of the 2022 IEEE International Conference on Communication, Networks and Satellite (COMNETSAT).

[38] Judge R., Choi F., Helmuth B. (2018). Recent Advances in Data Logging for Intertidal Ecology. Front. Ecol. Evol..

[39] Siriwardana C., Cooray A.T., Liyanage S.S., Koliyabandara S.M.P.A. (2019). Seasonal and Spatial Variation of Dissolved Oxygen and Nutrients in Padaviya Reservoir, Sri Lanka. J. Chem..

[40] Vinson N.S., Ante S.C., Roxas R.J.F.S., Salvio S.M.C., Rabe S.L.C., Torres M.A.J., Requieron E.A. (2016). Correlation between water quality and seagrass distribution along intertidal zone in Sarangani Province, Philippines. J. Biodiv. Environ. Sci..

[41] Conaco C., Cabaitan P.C. (2020). Influence of salinity and temperature on the survival and settlement of Heliopora coerulea larvae. Mar. Pollut. Bull..

[42] Faizal I., Purba N.P. *AWS Dataset Pangandaraan*; Mendeley Data, V4; 2023. https://data.mendeley.com/datasets/w3ptrd25yt/4.

[43] Firing E., Hummon J., Chereskin T. (2012). Improving the Quality and Accessibility of Current Profile Measurements in the Southern Ocean. Oceanography.

[44] Auraen J. *Low-Cost CTD Instrument Arduino Based CTD for Autonomous Measurement Platform. Thesis Report*, Oslo, Norway 2019. p. 75. https://www.duo.uio.no/bitstream/handle/10852/68775/1/Low-cost-CTD-Instrument---Arduino-based-CTD-for-autonomous-measurement-platform.pdf.

[45] Lockridge G., Dzwonkowski B., Nelson R., Powers S. (2016). Development of a Low-Cost Arduino-Based Sonde for Coastal Applications. Sensors.

[46] Medina J.D., Arias A., Triana J.M., Giraldo L.F., Segura-Quijano F., Gonzalez-Mancera A., Zambrano A.F., Quimbayo J., Castillo E. (2022). Open-source low-cost design of a buoy for remote water quality monitoring in fish farming. PLoS ONE.

[47] König C., Helmi A.M. (2020). Sensitivity Analysis of Sensors in a Hydraulic Condition Monitoring System Using CNN Models. Sensors.

